# Integrating Focused Assessment With Sonography for Trauma (FAST) Ultrasound Training Into the Osteopathic Medical Student – Year 1 (OMS-I) Curriculum: A Prospective Observational Study of Student Perceptions and Confidence

**DOI:** 10.7759/cureus.108097

**Published:** 2026-05-01

**Authors:** Chad Tomassetti, Hannah G VanHoozier, Joel Divaker, Nadiya A Persaud, Linda Brecher

**Affiliations:** 1 Research, Orlando College of Osteopathic Medicine, Winter Garden, USA; 2 Public Health, University of South Florida, Tampa, USA

**Keywords:** clinical readiness, fast, fast exam, first-year medical students, focused assessment with sonography for trauma, hands-on training, medical education, pocus, point-of-care ultrasound

## Abstract

Introduction

Point-of-care ultrasound (POCUS) has seen rapid growth in recent years and has become a key part of everyday diagnostic assessments in clinical practice. With the growing concern over the frequency of unnecessary testing in hospitalized patients, often leading to increased discomfort, potential harm, and rising healthcare costs, POCUS offers a practical solution by providing immediate bedside evaluation that can reduce reliance on more invasive or redundant diagnostic procedures. Because of this, medical schools have started to incorporate POCUS training into their curriculum.

Objective

First-year medical students (OMS-1) at the Orlando College of Osteopathic Medicine met to form the Ultrasound Club, an interest group to gain familiarity with and early experience in ultrasound. As part of this initiative, a physician-led Focused Assessment with Sonography in Trauma (FAST) exam workshop was conducted. The objective of this study is to evaluate students' knowledge, perceptions, and confidence levels before and after the intervention, using pre- and post-workshop surveys. It was hypothesized that students would demonstrate improvements in self-reported knowledge and confidence with ultrasound following the workshop.

Methods

Twenty-nine OSM-1s participated in a physician-led FAST exam workshop. The session included a live demonstration and guided hands-on practice. Students completed pre- and post-workshop surveys assessing self-reported knowledge, confidence, and attitudes on a seven-point Likert scale.

Results

A total of 29 students attended the workshop; all 29 completed the pre-workshop survey, and 26 completed the post-workshop survey. Results showed that the students overall felt more confident and competent in their ability to perform the FAST exam. Student interest and support for continued ultrasound education increased from the pre-workshop survey to the post-workshop survey.

Conclusion

Early ultrasound training was associated with improvements in medical students’ self-reported confidence, interest, and perceived ultrasound-related skills. These findings support the integration of ultrasound into preclinical osteopathic curricula to reinforce anatomy and promote hands-on, holistic care.

## Introduction

Point-of-care ultrasound (POCUS) is becoming increasingly accessible and widely adopted across a range of specialties, including emergency medicine, sports medicine, obstetrics and gynecology, and anesthesia, due to its affordability, convenience, and noninvasive nature [1,2]. Its real-time imaging capabilities support faster clinical decision-making and reduce reliance on more costly or time-consuming diagnostic tools. As a result, POCUS is often regarded as a valuable extension of the physical examination [3].

Because of the increasing relevance of ultrasound in clinical practice, many medical schools, and even some undergraduate pre-medical programs, have incorporated ultrasound education into their preclinical curriculum [1,2]. Studies have demonstrated that ultrasound training not only improves students’ proficiency in image acquisition and interpretation but also enhances their understanding of anatomy and physiology [3]. Analysis based on pre- and post-intervention surveys consistently shows improvement in both knowledge and confidence [4]. Since ultrasound accuracy is highly dependent on the skill of the operator, it is beneficial to train students early and often to reduce diagnostic errors and better prepare future residents and attending physicians [5]. Research supports the effectiveness of both faculty-led and student-led instruction models for ultrasound education [6-9]. Success from these events in various school settings suggests that those benefits can be reproduced [1,10,11]. Curricula have covered specialties such as cardiology, pulmonology, gastroenterology, genitourinary, and musculoskeletal systems, typically employing a structured approach that includes pre-assessment, instructional videos, didactic lectures, hands-on practice sessions with instructors, and post-assessment surveys. Beyond education, integrating POCUS into clinical assessments has been shown to reduce unnecessary and redundant testing, which otherwise contributes to escalating healthcare costs and potential patient harm. Integrating POCUS into routine diagnostic assessments may help mitigate this issue by offering immediate insight at the bedside, reducing reliance on more invasive or excessive testing [12]. Improving ultrasound education early in medical training could thus influence future imaging order practices, potentially reducing unnecessary testing. 

The diagnostic advantages of POCUS compared to physical examination alone are well-documented. In one study, first-year medical students with minimal ultrasound exposure achieved a 75% diagnostic accuracy rate for valvular disease, left ventricular dysfunction, enlargement, and hypertrophy using handheld ultrasound devices, compared to a 49% accuracy rate by board-certified cardiologists performing cardiac physical examinations [13]. In a similar study measuring liver size by physical examination, the medical students were once again more accurate compared to board-certified internists [14]. Hands-on ultrasound training not only enhances immediate diagnostic skills but also promotes long-term retention of anatomy knowledge, increases confidence, and fosters greater comfort in clinical settings [15]. Since ultrasound is the most frequently used imaging modality, training will help bridge the gap between medical education and everyday clinical skills [16]. 

One essential clinical application of POCUS is the Focused Assessment with Sonography in Trauma (FAST) exam, a rapid bedside ultrasound protocol used to identify free fluid in trauma patients. The FAST exam evaluates the pericardium as well as three key areas of the abdominal cavity: the right upper quadrant (RUQ), left upper quadrant (LUQ), and suprapubic region [17]. The utilization of the FAST exam in emergency settings has been very helpful in quickly and reliably identifying the presence of free fluid in the body that needs to be addressed as soon as possible [18]. Although this diagnostic exam can be extremely helpful in quick diagnosis of potentially life-threatening internal injuries, the FAST exam requires a level of mastery with the POCUS equipment. Data shows that formal ultrasound training is often limited or introduced late in medical education, leaving resident and attending physicians lacking the confidence in their POCUS skills to actually continue using it in their daily practice. Early ultrasound exposure reinforces both anatomical knowledge and procedural skills, which aligns particularly well with the hands-on, patient-centered philosophy of osteopathic medicine [1].

In response to these challenges and opportunities, we implemented a physician-led FAST ultrasound workshop for first-year students at the Orlando College of Osteopathic Medicine. This study aims to evaluate the impact of the workshop on students’ self-reported confidence, perceived competence, and motivation to incorporate ultrasound into their future clinical practice.

This work was previously presented as a poster at the Orlando College of Osteopathic Medicine Research Symposium on April 4, 2025.

## Materials and methods

Study design and setting

This prospective observational study was conducted at the Orlando College of Osteopathic Medicine (OCOM), Florida, US, during the 2025 spring semester. The study evaluated the impact of a physician-led FAST ultrasound workshop on first-year medical students’ knowledge, confidence, and perceptions. The FAST examination was taught using a standardized four-window approach, including the RUQ (Morison’s pouch), subcostal cardiac, LUQ (splenorenal), and pelvic views, with emphasis on identifying key anatomical landmarks and proper probe positioning during hands-on instruction [19,20].

**Figure 1:**
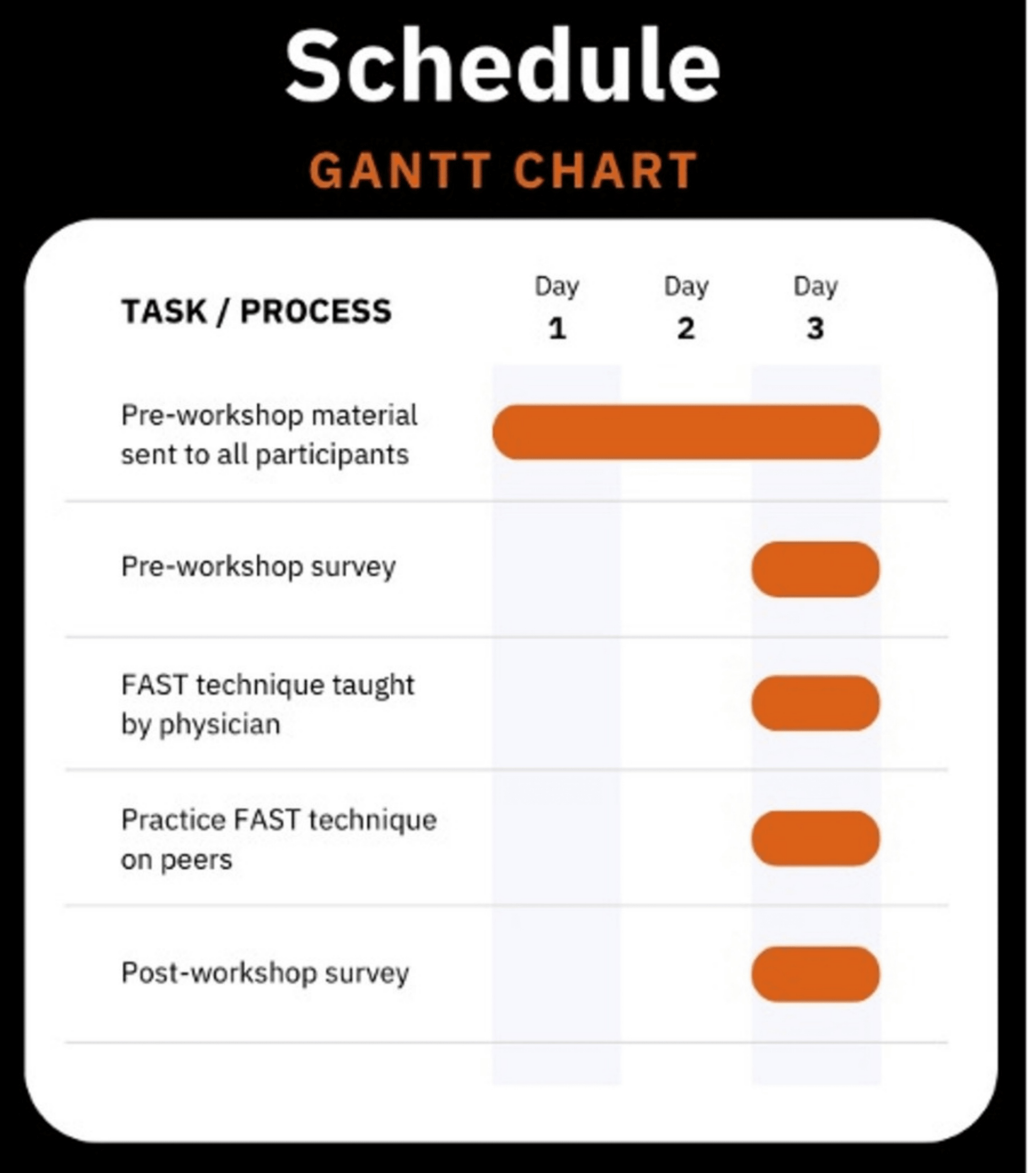
Gantt chart depicting the timeline of pre-workshop preparation, instruction, and assessment This Gantt chart outlines the three-day timeline of activities associated with the Focused Assessment with Sonography in Trauma (FAST) exam workshop. The chart illustrates the sequencing and duration of each task to clarify the structure of the educational intervention. Image credit: Created by VanHoozier H, using Canva (Canva Inc., Perth, Australia).

A visual timeline of the workshop workflow, including distribution of preparatory materials, survey administration, and instructional components, is presented in Figure 1. 

Participants were provided with pre-workshop instructional materials consisting of externally sourced educational digital resources reviewing FAST exam principles, probe handling, and relevant anatomical landmarks. The pre-workshop survey was administered on the day of the session, followed by the physician-led instruction on FAST examination techniques and guided hands-on practice. The post-workshop survey was completed at the conclusion of the training session.

Inclusion and exclusion criteria

All first-year osteopathic medical students (OMS-1) enrolled at the institution were eligible to participate in the study. Inclusion criteria consisted of voluntary attendance at the workshop and completion of at least one survey (pre- or post-workshop). There were no formal exclusion criteria; however, students who did not attend the workshop or did not complete any portion of the survey were not included in the analysis.

Participants and recruitment

All 96 OMS-1s were invited to participate in the workshop, and 29 elected to attend. Upon arrival, participants signed waivers and completed a pre-workshop survey prior to beginning the instructional session.

Workshop structure and educational intervention

The workshop was led by a board-certified emergency medicine physician and supported by two additional physicians (emergency medicine and rheumatology) who circulated among groups to facilitate hands-on learning. The session included a didactic lecture outlining essential FAST exam views and their clinical relevance, followed by guided ultrasound practice. The workshop lasted two hours. Approximately 30 minutes were dedicated to didactic instruction, followed by 90 minutes of hands-on practice with real-time instructor feedback.

Instructional materials and pre-workshop preparation

To support foundational learning, participants were provided instructional ultrasound videos via email three days before the workshop. These materials reviewed FAST exam principles, probe handling, and anatomical landmarks.

Hands-on training procedures

Upon arrival, students signed up for small groups, ensuring no more than four participants were assigned per ultrasound machine. Each group selected a volunteer “patient,” and students rotated roles to allow hands-on practice acquiring all four standard FAST exam views. Facilitators provided real-time feedback on image acquisition, probe orientation, and anatomical identification throughout the session.

Survey instruments and data collection

Pre- and post-workshop surveys were administered to assess changes in students’ confidence, knowledge, and perceptions. Data collection was conducted using structured electronic survey instruments distributed to participants before and immediately after the workshop session. The survey instrument was developed by the study authors and consisted of items measured on a seven-point Likert scale (1 = strongly disagree, 7 = strongly agree). The questionnaire included both quantitative Likert-scale items and qualitative open-ended questions to capture participant experiences and feedback (Table 1).

**Table 1:** Ultrasound workshop survey instrument OCOM: Orlando College of Osteopathic Medicine.

Section	Item #	Survey Item
Quantitative (Likert Scale)	1	I feel confident applying proper probe techniques for FAST (Focused Assessment with Sonography for Trauma) exams.
	2	I am confident identifying and interpreting the relevant anatomical structures with ultrasound.
	3	I feel comfortable performing a FAST exam on my own.
	4	I feel comfortable teaching someone else how to perform a FAST exam.
	5	I am enthusiastic about integrating point-of-care ultrasound in my future practice.
	6	I support continued growth and integration of ultrasound technology at OCOM.
	7	I intend to continue learning about ultrasound and refining my skills beyond this workshop.
	8	I am interested in becoming a peer-to-peer ultrasound instructor next year.
	9	I felt that I had sufficient time to practice and acquire the necessary ultrasound images.
	10	The group size in this workshop was appropriate for effective hands-on learning.
	11	The workshop content was a valuable supplement to my anatomy studies.
Qualitative (Open-Ended)	12	What motivated you to attend this ultrasound workshop, and what do you hope to learn or achieve? What interests you about ultrasound?
	13	What did you like most about this workshop? Which part(s) of the workshop (e.g., lecture, hands-on scanning, Q&A) did you find most valuable, and why?
	14	What aspects of the workshop did you find challenging or would you change, and why?
	15	What suggestions do you have for improving future ultrasound workshops?

The instrument was not previously validated nor formally assessed for reliability. Open-ended questions were also included to capture qualitative feedback regarding workshop strengths, challenges, and recommendations for future sessions.

Quantitative data analysis

Survey data were exported and analyzed using RStudio (Posit, Boston, MA) with R version 4.4.1 (R Foundation for Statistical Computing, Vienna, Austria, https://www.R-project.org/). Descriptive statistics were generated for all Likert-scale items. Pre- and post-workshop trends were examined to evaluate changes in knowledge, confidence, and perceived preparedness. Given the small sample size and study design, analyses were descriptive in nature, and no inferential statistical tests were performed to assess statistical significance. Changes in the Likert-scale responses were evaluated using summary statistics and observed trends. Pearson correlation coefficients were calculated to assess relationships among confidence in performing the FAST exam, identifying anatomical structures, applying proper probe techniques, and teaching the exam to others. Given the small sample size and exploratory nature of the study, analyses were descriptive in nature, and no inferential statistical tests (e.g., paired t-tests) were performed.

Qualitative data analysis

Responses to the open-ended questions were reviewed and coded to identify recurring themes related to participant satisfaction, perceived strengths of the workshop, areas for improvement, and suggestions for future ultrasound training opportunities. Qualitative analysis was conducted using an inductive thematic approach, in which responses were independently reviewed and grouped into thematic categories based on recurring patterns in the data. Themes were then refined through consensus among the study authors.

## Results

Survey completion and response rates

A total of 29 students attended the workshop. Survey completion and response rates are summarized in Table 2.

**Table 2:** Survey completion and response rates

Measure	Value
Total workshop participants	29
Pre-workshop survey responses	29 (100%)
Post-workshop survey responses	26 (89.7%)
Survey attrition	3 (10.3%)

Pre-workshop survey responses were obtained from 29 (100%) participants, while 26 (89.7%) participants completed the post-workshop survey, reflecting a small attrition of three students (10.3%). A summary of survey completion and response rates is presented in Table 2. Across all measured domains, post-workshop scores demonstrated improvement, reflecting increased confidence, enthusiasm, and perceived competence in performing the FAST exam.

Pre-workshop perceptions and correlation analysis

Analyses revealed strong associations between several domains of perceived competence before the workshop. These findings are summarized in Table 3.

**Table 3:** Pre-workshop correlation analysis of perceived FAST exam competencies FAST: Focused assessment with sonography for trauma.

Variable Pair	Correlation Coefficient (r)
Performing FAST vs Teaching FAST	0.83
Performing FAST vs Identifying anatomy	0.65
Performing FAST vs Probe technique	0.64
Teaching FAST vs Identifying anatomy	0.77
Teaching FAST vs Probe technique	0.69

Confidence in performing the FAST exam was closely related to confidence in teaching others, identifying anatomical structures, and applying proper probe techniques, with correlation coefficients of 0.83, 0.65, and 0.64, respectively. Similarly, confidence in teaching the FAST exam correlated strongly with the ability to identify anatomical structures (r=0.77) and applying appropriate probe techniques (r=0.69). These relationships suggest that even prior to formal instruction, students viewed these skills as interconnected and foundational components of effective ultrasound performance.

Post-workshop changes in confidence and perceptions

Following the workshop, students reported notable increases in confidence across all domains. Post-workshop changes are summarized in Table 4.

**Table 4:** Post-workshop correlations and changes in confidence and perceptions Related competencies include identifying anatomical structures, applying proper probe techniques, performing the FAST exam independently, and teaching the exam to others. FAST: Focused assessment with sonography for trauma.

Variable Pair	Correlation Coefficient (r)
Performing FAST vs Related competencies	0.88
Teaching FAST vs Related competencies	0.80
Enthusiasm vs Curriculum support	0.95

Confidence in performing and teaching the FAST exam improved substantially, with correlations of 0.88 and 0.80 observed across related competencies, including anatomical identification, probe technique, independent performance, and teaching ability, with correlations of 0.88 and 0.80 observed across related skill sets. Enthusiasm for ultrasound and support for continued integration of POCUS within the OCOM curriculum were also high, with correlation values of 0.95. Students expressed a strong desire to continue learning ultrasound beyond the workshop and reported high satisfaction with the hands-on practice time provided. These findings indicate that the workshop effectively strengthened perceived competence and broader attitudes toward ultrasound education.

Qualitative feedback and thematic impressions

Narrative responses further highlighted the workshop’s educational impact. A thematic summary of qualitative feedback is presented in Table 5.

**Table 5:** Thematic analysis of qualitative feedback

Theme	Description	Representative Feedback
Hands-on learning	Value of direct scanning experience	“Hands-on practice was the most helpful part of the workshop.”
Instructor guidance	Benefit of real-time feedback	“Having instructors guide us improved my understanding significantly.”
Reinforcement of anatomy	Improved anatomical understanding	“It helped connect anatomy to real clinical imaging.”
Desire for more practice	Need for extended time	“More hands-on time would improve the experience.”
Group size considerations	Preference for smaller groups	“Smaller groups would allow more individualized learning.”

Students emphasized the value of hands-on scanning, personalized instructor guidance, and the opportunity to reinforce anatomical knowledge through practical application. Suggestions for improving future sessions included increasing hands-on practice time, reducing group sizes to allow more individualized instruction, and incorporating additional guided demonstrations at the start of the workshop.

Overall impact

**Figure 2:**
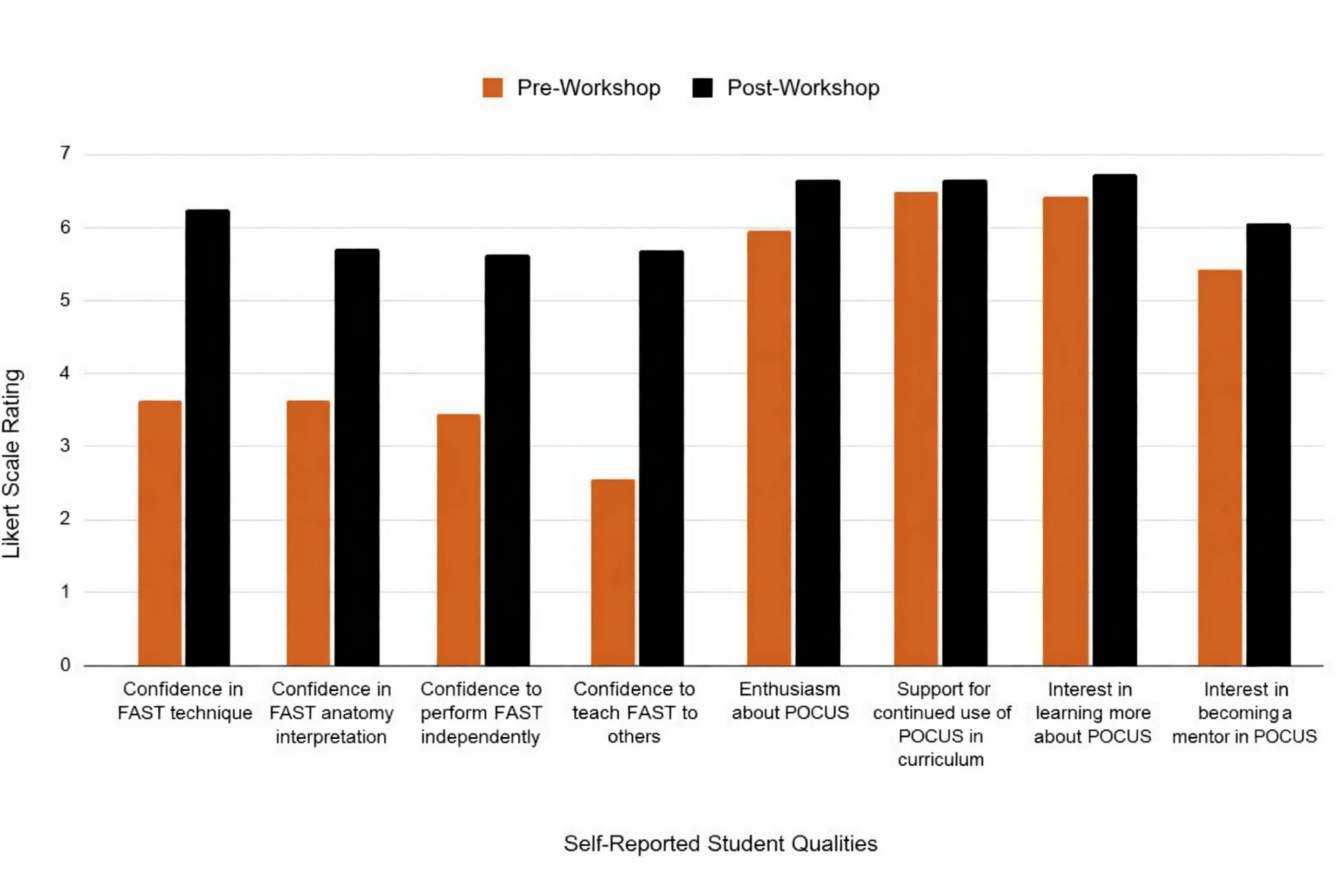
Pre- and post-workshop survey results demonstrating changes in self-reported student confidence, competence, and interest in POCUS Bar graph comparing pre- and post-workshop Likert-scale ratings across eight domains related to focused assessment of sonography for trauma (FAST) exam proficiency and interest in point-of-care ultrasound (POCUS). Image credit: Created by VanHoozier H, using Canva (Canva Inc., Perth, Australia).

Overall, the results demonstrate that even a single, focused FAST exam workshop can produce meaningful gains in self-reported competence and enthusiasm for ultrasound among first-year medical students. The overwhelmingly positive feedback and consistent increases across all confidence-related measures support the feasibility and educational value of early, hands-on ultrasound exposure within the preclinical curriculum. As shown in Figure 2, mean Likert-scale ratings increased across all domains, demonstrating marked improvements in students’ confidence, perceived competence, and enthusiasm for ultrasound following the workshop. 

Students reported improvements in confidence with FAST technique, anatomical interpretation, independent performance, and teaching ability following the workshop. Enthusiasm for POCUS, support for continued POCUS integration in the curriculum, interest in further learning, and interest in future mentorship roles also increased. Higher scores reflect greater agreement with each survey item.

## Discussion

POCUS has implications beyond education, with growing evidence supporting its role in improving clinical efficiency and reducing unnecessary diagnostic testing [12]. Early exposure to ultrasound during medical training may therefore not only enhance student confidence but also influence future clinical decision-making. Integrating structured ultrasound training into preclinical curricula may help bridge the gap between foundational science education and real-world clinical application.

This study demonstrates that early exposure to POCUS, specifically through a FAST exam workshop, is associated with improvements in first-year medical students’ self-reported confidence, perceived competence, and appreciation for ultrasound. Participation in a single, structured workshop was associated with increased confidence and a positive overall response. While improvements in confidence following instruction are expected, these findings highlight the practical value of brief, targeted educational interventions. In the context of increasingly dense medical curricula, such workshops offer a feasible and scalable approach to introducing emerging clinical skills without requiring extensive curricular restructuring.

The observed increases in post-workshop survey scores suggest that even short-duration ultrasound training can have a meaningful impact on early medical education. Participants reported increased confidence in ultrasound-related skills, greater comfort teaching the material to peers, and heightened enthusiasm for incorporating ultrasound into future clinical practice. These findings underscore a growing demand for early integration of ultrasound education within medical curricula.

The results align with broader trends in medical education that emphasize early clinical exposure and the integration of technology into bedside practice. The workshop model used in this study, characterized by a small instructor-to-student ratio, portable ultrasound devices, and focused hands-on learning, demonstrates both feasibility and scalability [21-24]. For example, a prospective study evaluating a brief POCUS training workshop reported significant gains in image acquisition, interpretation, and procedural confidence following a combined didactic and hands-on session. Additionally, early integration of ultrasound into preclinical curricula has been associated with increased student interest and improved understanding of anatomy and clinical application. The results of the present study align with this body of literature, further supporting the value of early, targeted ultrasound exposure in medical education [24]. Incorporating ultrasound training into preclinical education may help bridge the transition from basic science learning to clinical application, ultimately supporting the development of more confident and prepared future physicians.

In osteopathic medical education, early ultrasound exposure aligns with the core principles of holistic, patient-centered care. By reinforcing anatomical understanding through real-time imaging and hands-on practice, ultrasound training complements osteopathic manipulative medicine (OMM) and enhances students’ appreciation for physical diagnosis.

An additional notable finding is the strong relationship between confidence in performing the FAST exam and confidence in teaching the skill to others. This suggests that early ultrasound exposure may support not only individual skill development but also peer-to-peer learning models, which are increasingly utilized in medical education.

Early exposure to POCUS during preclinical education may play a critical role in shaping students’ familiarity with bedside diagnostic tools. Integrating ultrasound at this stage allows students to connect foundational anatomical knowledge with real-time imaging, potentially improving retention and facilitating smoother transitions into clinical training. These findings contribute to ongoing discussions regarding how best to incorporate new technologies into medical education. Focused workshops may serve as an efficient entry point for institutions seeking to expand ultrasound training within existing curricular constraints.

This study has several limitations. The small sample size and reliance on self-reported data may introduce bias. The use of subjective measures without objective assessment of technical skill limits the ability to draw conclusions regarding true skill acquisition. Additionally, the absence of a control group restricts causal inference, and the study design captures only immediate post-intervention effects without evaluating long-term retention. The participation rate was relatively low (29 of 96 eligible students), and voluntary attendance introduces potential selection bias, as participants may have had a pre-existing interest in ultrasound and may have been more likely to report favorable outcomes. Furthermore, the survey instrument was not previously validated nor formally assessed for reliability, which may affect the consistency of the findings. Additionally, inferential statistical comparisons between pre- and post-workshop responses were not performed, which limits the ability to formally assess statistical differences between time points.

Future research should incorporate objective assessments of skill acquisition, evaluate long-term retention, and explore the impact of early ultrasound training on clinical performance during later stages of medical education. Continued investigation into POCUS integration will be essential as ultrasound becomes an increasingly central component of modern clinical practice.

## Conclusions

This study demonstrates that early, hands-on exposure to the FAST ultrasound exam is associated with increases in self-reported confidence, perceived ultrasound-related skills, and interest among OMS-I. For osteopathic students in particular, early ultrasound education reinforces the philosophy of holistic, hands-on patient care that is central to osteopathic medicine. The workshop format of combining live demonstration with guided peer practice proved effective in enhancing student engagement and confidence in ultrasound-related tasks. Furthermore, student enthusiasm for integrating POCUS into future clinical practice increased, highlighting the value of introducing ultrasound training early in medical education. These findings support the continued development and expansion of ultrasound curricula to better equip future physicians for clinical training, while recognizing that further research with objective outcome measures is needed to assess skill acquisition and long-term impact.
